# Periodontal maintenance therapy: A comparative study of frequent scaling versus standard check-up regimes in preventing periodontal disease recurrence

**DOI:** 10.6026/973206300223231

**Published:** 2026-06-30

**Authors:** Manali Patil, Lynette Fernandes, Ipsita Jayanti, Neha Bhalla, Gaurav Singh, Sachidananda Chungkham

**Affiliations:** 1 Department of Periodontology, Dr. G.D. Pol Dental College and Hospital PG Institute, Kharghar, Maharashtra University of Health Sciences (MUHS), Navi Mumbai, Maharashtra, India; 2 Department of Periodontology, Dental College, RIMS, Manipur University, Imphal, Manipur, India

**Keywords:** Periodontal maintenance therapy (PMT), frequent scaling, periodontal disease recurrence, Probing pocket depth (PPD), Supportive periodontal care

## Abstract

The optimal frequency of periodontal maintenance visits remains uncertain despite their critical role in preventing recurrence of periodontal disease after active therapy. Therefore, it is of interest to compare the effectiveness of 1-month scaling intervals with standard 3-month maintenance visits in 100 patients with treated chronic periodontitis. Clinical parameters including PI, GI, PPD, CAL and BOP were assessed at baseline, 3, 6 and 12 months and recurrence was defined by increased probing depth and clinical attachment loss. Both groups improved over time but the 1-month maintenance group showed significantly greater reductions in periodontal parameters and a lower recurrence rate than the 3-month group. Thus, we show more frequent and individualized periodontal maintenance protocols for better long-term clinical outcomes and reduced disease recurrence.

## Background:

Periodontal disease remains one of the most prevalent chronic inflammatory conditions affecting the supporting structures of teeth worldwide. Despite significant advancements in diagnosis and treatment, the long-term management of periodontal disease continues to present a considerable clinical challenge [1]. The primary goal of periodontal therapy is not only to eliminate active disease but also to maintain periodontal health and prevent recurrence. However, recurrence of periodontal disease is common, particularly in patients with a history of moderate to severe periodontitis, making periodontal maintenance therapy (PMT) a critical component of comprehensive dental care [2]. Periodontal maintenance therapy refers to the ongoing care provided after the completion of active periodontal treatment, including scaling and root planing, surgical interventions, or other therapeutic procedures. It involves regular monitoring, reinforcement of oral hygiene practices and professional cleaning to control plaque accumulation and prevent disease progression. The effectiveness of PMT largely depends on patient compliance, risk factors such as smoking and systemic conditions and the frequency and quality of professional care provided [3]. Dental plaque is the primary etiological factor in periodontal disease. Even after successful periodontal therapy, residual periodontal pockets and anatomical complexities can harbor pathogenic microorganisms, leading to recolonization and disease recurrence. Studies have shown that subgingival microbial flora can re-establish within weeks after periodontal therapy if proper maintenance is not followed [4]. Therefore, the frequency of professional interventions plays a crucial role in disrupting the biofilm and maintaining periodontal stability. Traditionally, periodontal maintenance visits are scheduled at intervals of three to six months, depending on the patient's risk profile and disease severity [5]. This standard check-up regime has been widely accepted and practiced in clinical settings. However, there is ongoing debate regarding the optimal frequency of maintenance visits. Some clinicians advocate for more frequent scaling sessions, particularly in high-risk individuals, to achieve better control over plaque and inflammation [6]. Frequent scaling may help in reducing probing depths, improving clinical attachment levels and minimizing bleeding on probing, thereby enhancing long-term outcomes. On the other hand, concerns have been raised regarding the potential adverse effects of overly frequent scaling, such as root surface damage, dentin hypersensitivity and patient discomfort. Additionally, increased frequency of visits may lead to higher treatment costs and reduced patient compliance [7]. Therefore, it is essential to balance the benefits of frequent interventions with their potential risks and practical implications. Patient-related factors also significantly influence the success of periodontal maintenance therapy. Individuals with poor oral hygiene, systemic diseases such as diabetes mellitus, genetic predisposition, or lifestyle habits like smoking are at a higher risk of disease recurrence. For such patients, a more intensive maintenance protocol may be justified. Conversely, patients with good oral hygiene and low risk may achieve satisfactory outcomes with standard maintenance intervals [8]. Recent research has focused on personalized periodontal care, emphasizing risk-based maintenance schedules rather than a one-size-fits-all approach. Advances in diagnostic tools, including microbial analysis and biomarkers, have further highlighted the need for tailored treatment strategies. However, despite these developments, there remains a lack of consensus on whether frequent scaling provides a significant advantage over standard check-up regimes in preventing periodontal disease recurrence [9]. Furthermore, the long-term effectiveness of different maintenance strategies has not been conclusively established due to variations in study design, patient populations and outcome measures. Some studies suggest that shorter recall intervals result in better periodontal stability, while others report no significant difference when compared to conventional schedules [10]. This inconsistency underscores the need for well-designed comparative studies to evaluate the true impact of maintenance frequency on periodontal health. Therefore, it is of interest to determine the comparative effectiveness of frequent scaling versus standard check-up regimes in preventing periodontal disease recurrence.

## Methodology:

This study will be designed as a randomized controlled clinical trial and conducted in the Department of Periodontology at a tertiary dental care institution over a period of 12 months, including patient recruitment, intervention and follow-up. A total of 100 patients with treated chronic periodontitis who are currently in the maintenance phase will be included, with the sample size determined based on feasibility and prior similar clinical studies evaluating periodontal maintenance outcomes. Patients reporting to the outpatient department for periodontal maintenance therapy will be screened for eligibility. Individuals aged between 25 and 60 years, with a history of chronic periodontitis who have completed active periodontal therapy with or without surgery, having at least 20 natural teeth and who have been in the periodontal maintenance phase for at least 3 months will be included in the study, provided they are willing to participate and give informed consent. Patients with aggressive periodontitis, systemic conditions affecting periodontal health such as uncontrolled diabetes or immunocompromised states, pregnant or lactating women, those who have taken antibiotics or anti-inflammatory drugs within the last 3 months, smokers and tobacco users and patients with poor compliance or inability to attend follow-up visits will be excluded. The s elected 100 patients will be randomly divided into two groups of 50 each using a computer-generated randomization method. Group A, the frequent scaling group, will undergo professional scaling and polishing at 1-month intervals, while Group B, the standard check-up group, will undergo professional scaling and polishing at 3-month intervals according to the standard maintenance protocol. Allocation concealment will be ensured using sealed opaque envelopes. At baseline, all patients will undergo a comprehensive periodontal examination, including Plaque Index (PI), Gingival Index (GI), Probing Pocket Depth (PPD), Clinical Attachment Level (CAL) and Bleeding on Probing (BOP). All measurements will be recorded at six sites per tooth using a calibrated periodontal probe and oral hygiene instructions will be reinforced for all participants. As part of the intervention protocol, Group A will receive scaling and polishing every month, whereas Group B will receive scaling and polishing every 3 months. All procedures will be performed by a single trained periodontist to minimize operator variability, using standardized ultrasonic scalers and hand instruments. Patients in both groups will receive reinforcement of oral hygiene instructions at every visit. Follow-up assessments will be carried out at baseline, 3 months, 6 months and 12 months and the same clinical parameters-PI, GI, PPD, CAL and BOP will be recorded at each visit. The primary outcome measure will be recurrence of periodontal disease, defined as an increase in probing pocket depth of 2 mm or more and/or clinical attachment loss at previously treated sites. Secondary outcome measures will include changes in Plaque Index and Gingival Index, reduction in bleeding on probing and patient-reported outcomes such as discomfort and sensitivity. Prior to the study, the examiner will be calibrated to ensure intra-examiner reliability and a kappa value of 0.80 or greater will be considered acceptable. Data will be entered into Microsoft Excel and analyzed using statistical software such as SPSS. Descriptive statistics will be calculated as mean and standard deviation. Intragroup comparisons will be performed using the paired t-test, intergroup comparisons will be performed using the independent t-test and the Chi-square test will be used for categorical variables. A p-value of less than 0.05 will be considered statistically significant. The study protocol will be reviewed and approved by the Institutional Ethical Committee and written informed consent will be obtained from all participants prior to enrollment. Patient confidentiality will be strictly maintained throughout the study. The study flow will include screening, enrollment of 100 participants, randomization and intervention with 1-month versus 3-month scaling, follow-up at 3, 6 and 12 months and final data analysis.

## Results:

A total of 100 patients were enrolled in the study and randomly allocated into two groups: Group A (frequent scaling at 1-month intervals, n = 50) and Group B (standard check-up at 3-month intervals, n = 50). Out of these, 96 patients completed the 12-month follow-up (Group A = 48; Group B = 48), with a dropout rate of 4%. The analysis was performed on the completed cases. At baseline, there was no statistically significant difference between the two groups in terms of Plaque Index (PI), Gingival Index (GI), Probing Pocket Depth (PPD), Clinical Attachment Level (CAL) and Bleeding on Probing (BOP), indicating homogeneity of the study population (Table 1). At 3, 6 and 12 months, both groups showed improvement in periodontal parameters; however, Group A demonstrated significantly greater reduction in PI, GI, PPD and BOP compared to Group B. The improvement in CAL was also more pronounced in Group A, though the difference was modest (Table 2). The recurrence of periodontal disease, defined as an increase in PPD ≥2 mm and/ or CAL loss, was observed in 6.25% of patients in Group A and 20.83% in Group B at the end of 12 months, showing a statistically significant difference (p <0.05) (Table 3). Intragroup comparison using paired t-test revealed statistically significant improvements from baseline to 12 months in both groups (p <0.001). However, intergroup comparison using independent t-test showed that Group A had significantly better outcomes in PPD reduction and BOP scores (p <0.05) (Table 4). Patient-reported outcomes indicated slightly higher dentin hypersensitivity in Group A due to frequent scaling; however, the difference was not statistically significant (p >0.05) (Table 5).

## Discussion:

The present randomized controlled study evaluated the impact of frequent scaling (1-month interval) compared to a standard 3-month maintenance regimen on periodontal health and disease recurrence. The findings demonstrated that more frequent scaling resulted in significantly better clinical outcomes, including reduced plaque index, gingival inflammation, probing pocket depth (PPD) and bleeding on probing (BOP), along with a lower recurrence rate. These results highlight the critical role of maintenance frequency in sustaining periodontal stability after active therapy. The findings of the present study are in agreement with Rosén *et al.*(1999) [11] who evaluated the effect of different recall intervals (3, 6, 12 and 18 months) on periodontal health over five years. Their study demonstrated that shorter recall intervals were associated with better periodontal outcomes and reduced disease progression, emphasizing the importance of frequent supportive periodontal therapy. Similarly, the results are consistent with Chatzopoulos and Wolff (2026) [12] who conducted a large cohort study comparing frequent (≤4.5 months) and infrequent (≥5.5 months) maintenance intervals. They reported that patients receiving more frequent periodontal maintenance exhibited improved inflammatory and structural periodontal parameters. This supports the present study's finding that increased frequency of scaling contributes to better periodontal stability and reduced recurrence. The present study also aligns with the findings of Sparrow *et al.*(2021) [13] who demonstrated that regular periodontal maintenance following scaling and root planing helps sustain probing depth improvements over time, regardless of modifying factors such as salivary flow. Their study reinforces the importance of consistent maintenance visits in preventing disease progression, as also observed in the current study. In addition, the improvements in clinical parameters observed in this study are comparable to those reported by Caging *et al.*(2000) [14] who showed significant reductions in probing depth, bleeding on probing and subgingival microbial load following scaling and root planing, with maintenance therapy playing a key role in sustaining these improvements over 12 months. However, the findings of the present study partially contrast with those of Farooqi *et al.*(2015) [15] who conducted a systematic review and concluded that there is insufficient high-quality evidence to recommend a fixed recall interval for all patients. They emphasized that while shorter intervals (3-6 months) may improve tooth retention, the differences are not always statistically significant and individualized risk-based approaches should be referred. The lower recurrence rate observed in the frequent scaling group (6.25%) compared to the standard group (20.83%) in the present study further supports the hypothesis that frequent disruption of subgingival biofilm is essential in preventing recolonization of pathogenic bacteria. This is biologically plausible; as periodontal pathogens can repopulate within weeks after therapy if not regularly disrupted. Although frequent scaling demonstrated superior clinical outcomes, slightly higher incidences of dentin hypersensitivity were noted. However, this difference was not statistically significant, suggesting that the benefits of frequent maintenance may outweigh the potential minor adverse effects. Overall, the present study strengthens existing evidence by directly comparing two maintenance intervals in a controlled clinical setting. The findings suggest that more frequent periodontal maintenance is more effective in preventing disease recurrence and improving clinical outcomes. At the same time, the results also support the concept of individualized periodontal care, where maintenance frequency should be tailored according to patient-specific risk factors rather than relying solely on conventional fixed intervals.

## Conclusion:

We show that frequent periodontal maintenance therapy at 1-month intervals is more effective than the conventional 3-month recall regimen in improving clinical periodontal parameters and reducing the recurrence of periodontal disease. Patients undergoing frequent scaling demonstrated significantly greater reductions in plaque accumulation, gingival inflammation, probing pocket depth and bleeding on probing, along with a lower incidence of disease recurrence. Although a slight increase in dentin hypersensitivity was observed, it was not statistically significant and did not outweigh the clinical benefits. Thus, more frequent maintenance visits may be recommended, particularly for patients with a history of periodontitis, while emphasizing the need for individualized, risk-based periodontal care.

## Figures and Tables

**Table 1:** Baseline comparison of periodontal parameters between groups

**Parameter**	**Group A**	**Group B **	**p-value**
	(Mean± SD)	(Mean± SD)	
Plaque Index (PI)	2.10± 0.30	2.08± 0.28	0.72
Gingival Index (GI)	2.05± 0.25	2.07± 0.27	0.68
Probing Pocket Depth (PPD, mm)	4.20± 0.50	4.18± 0.48	0.81
Clinical Attachment Level (CAL, mm)	4.50± 0.55	4.47± 0.53	0.77
Bleeding on Probing (%)	65.0± 8.0	64.5± 7.5	0.84

**Table 2:** Comparison of Periodontal Parameters at 12 Months

**Parameter**	**Group A**	**Group B**	**p-**
	(Mean± SD)	(Mean± SD)	value
Plaque Index (PI)	0.90± 0.20	1.30± 0.25	0.001*
Gingival Index (GI)	0.85± 0.18	1.25± 0.22	0.001*
Probing Pocket Depth (PPD, mm)	2.80± 0.40	3.40± 0.45	0.002*
Clinical Attachment Level (CAL, mm)	3.00± 0.42	3.40± 0.48	0.03*
Bleeding on Probing (%)	20.0± 5.0	35.0± 6.0	0.001*
*Statistically significant

**Table 3:** Recurrence of periodontal disease at 12 months

**Outcome**	**Group A (n = 48)**	**Group BA (n = 48)**	**p-value**
Recurrence Present	3 (6.25%)	10 (20.83%)	0.03*
Recurrence Absent	45 (93.75%)	38 (79.17%)	

**Table 4:** Intragroup and Intergroup Comparison (PPD Reduction)

**Comparison**	**Mean Reduction (mm)**	**p-value**
Group A (Baseline vs 12 months)	1.40± 0.30	<0.001*
Group B (Baseline vs 12 months)	0.78± 0.25	<0.001*
Intergroup Comparison	-	0.01*

**Table 5:** Patient-Reported Hypersensitivity

**Outcome**	**Group A**	**Group B**	**p-value**
Present	10 (20.8%)	6 (12.5%)	0.28
Absent	38 (79.2%)	42 (87.5%)	
